# Influence of Ethnicity, Gender and Answering Mode on a Virtual Point-to-Origin Task

**DOI:** 10.3389/fnbeh.2016.00022

**Published:** 2016-02-23

**Authors:** Alexandra Kitson, Daniel Sproll, Bernhard E. Riecke

**Affiliations:** ^1^iSpace Lab, School of Interactive Arts and Technology, Simon Fraser UniversitySurrey, BC, Canada; ^2^Department of Neurobiopsychology, Institute of Cognitive Science, University of OsnabrückOsnabrück, Germany

**Keywords:** spatial navigation, reference frames, path integration, navigational strategies, gender differences, ethnicity differences

## Abstract

In a virtual point-to-origin task, participants seem to show different response patterns and underlying strategies for orientation, such as “turner” and “non-turner” response patterns. Turners respond as if succeeding to update simulated heading changes, and non-turners respond as if failing to update their heading, resulting in left-right hemisphere errors. We present two other response patterns, “non-movers” and “spinners,” that also appear to result in failures to update heading. We have three specific goals in mind: (1) extend previous findings of higher turner rates with spatial language response mode using a point-to-origin task instead of a triangle completion task; (2) replicate the gender effect of males more likely responding as turners; (3) examine ethnicity influence. Designed as a classroom study, we presented participants (*N* = 498) with four passages through a virtual star field. Participants selected the direction pointing to the origin from four multiple-choice items. Response mode was either pictograms or written language, chosen to compare with similar studies and see if these response modes have an effect on virtual orientation behavior. Results show a majority of participants (48.35%) classified as non-turners, 32.93% turners, 15.57% as non-movers, and 3.14% as spinners. A multinomial regression model reached 49% classification performance. Written spatial language, compared to pictograms, made turner response patterns more likely; this effect was more pronounced for Chinese participants and among females, but not male Caucasians. Moreover, higher turner numbers for written spatial language extends Avraamides findings of higher turner numbers when participants turned their bodies toward the origin but not when they responded verbally. Using pictorial response mode (i.e., top-down picture of a head) may have increased cognitive load because it could be considered more embodied. It remains to be seen how we can reduce the reference frame conflict that might have caused increased cognitive load. Second, our results are inconsistent with previous research in that males overall did not show more turner behavior than females. Future research may look at possible underlying factors, such as cultural norms. Third, individualistic cultures (Caucasians; Greif, 1994) lean toward turner response patterns, whereas collectivist cultures (Asian) lean toward non-turner response patterns.

## 1. Introduction

We are able to navigate and orient ourselves fairly effortlessly through the world. Yet, when we put ourselves in a virtual world, navigation often becomes cognitively more demanding. Why the discrepancy between real world navigation and virtual navigation? Normally we rely on vision, audition, vestibular, and proprioceptive cues to help guide us and update our position. In virtual reality (VR), however, physical motion cues and proprioceptive cues are often missing.

Spatial navigation is a deep rooted and modularized cognitive skill based on spatial representations that are automatically formed and maintained (updated) in specialized brain areas based on multimodal sensory information. Different reference frames for spatial orientations seem to be processed in distinct neural correlates (Zaehle et al., 2007; Gramann et al., 2010). The sensory information from all senses is automatically combined into a spatial representation in the brain involving a wide network of brain regions (for a review see Moser et al., 2008). However, there are times when spatial updating fails, especially when we receive incomplete or contradicting sensory information. In such cases, we often revert to offline strategies where we try to cognitively restore our spatial representations. Comparing online updating vs. offline strategies enables researchers to study the mechanism of spatial updating in more detail: when is spatial updating automatic and obligatory, and when does it brake down (Rieser, 1989; Farrell and Robertson, 1998; Riecke et al., 2005)? What factors decide which reference frame we use for solving a given task? Deepening our understanding of the factors that influence spatial representation and updating can enable us to apply these concepts in virtual environments to simulate self-motion and create a more realistic and natural sense of being in and moving through the virtual space, ultimately enabling unencumbered spatial orientation and performance.

While navigating, we use two distinct reference frames: egocentric, self-to-object representation, and allocentric, object-to-object representation (Klatzky, 1998). Forming and maintaining spatial representations is hard to suppress, and ignoring it takes conscious cognitive effort (Farrell and Robertson, 1998; Riecke et al., 2005). Yet, when we imagine navigating a path, we tend to use a mixed strategy to determine where we are. During navigation, spatial representations are not only constantly updated and maintained in parallel, but also interact (Moser et al., 2008). When exactly we use a specific reference frame for a certain task remains a difficult question, as the choice is not only based on task demands and available stimulus characteristics, but also affected by individual proclivities (Riecke, 2008; Goeke et al., 2013; Gramann, 2013).

**Figure 1:**
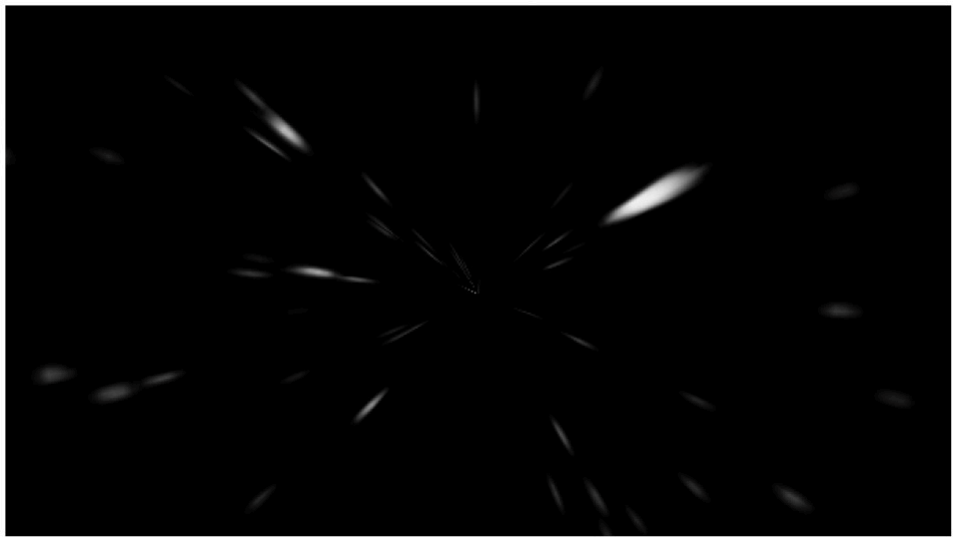
**A frame from the task here depicts moving through a star field**.

**Figure 2:**
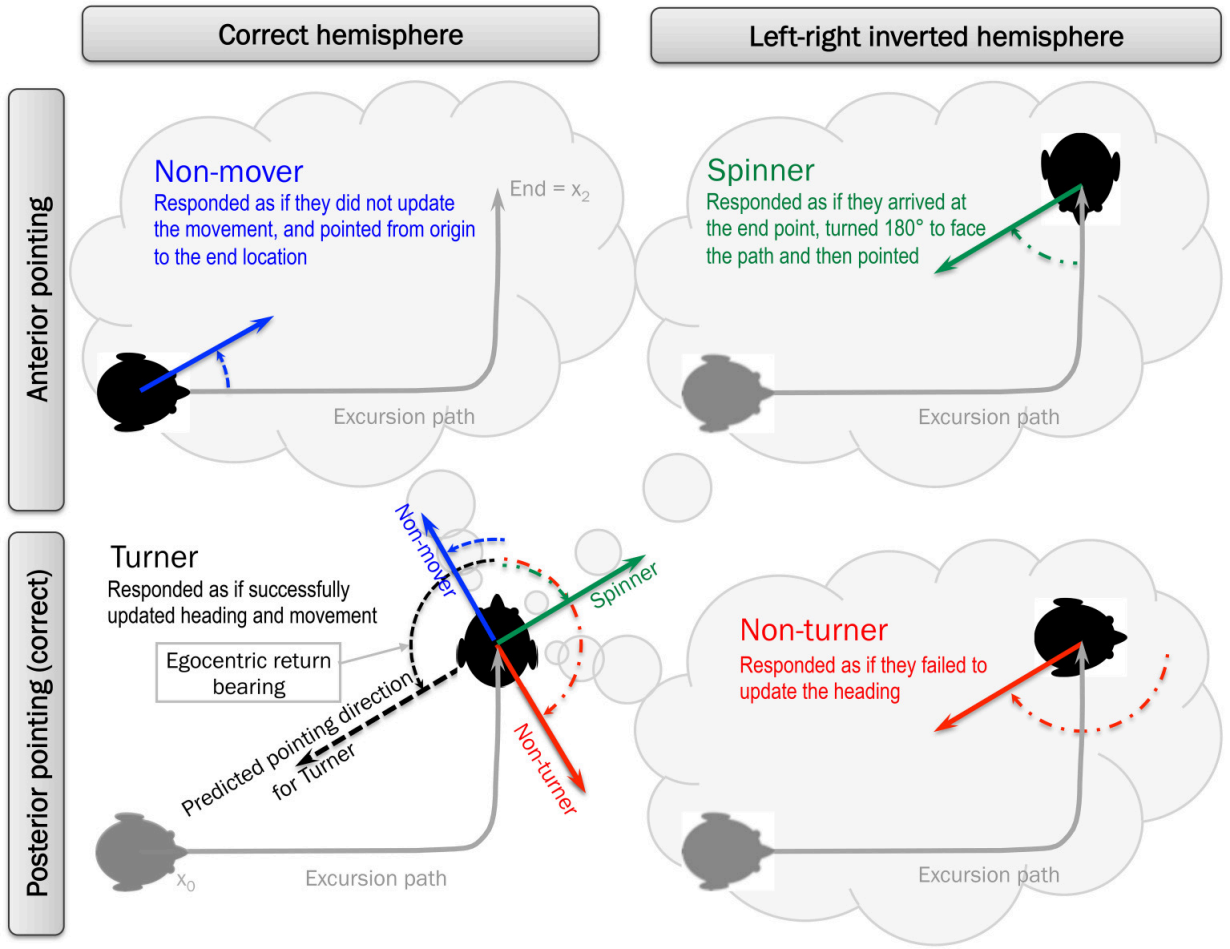
**Trajectories and predicted response patterns for turners (bottom-left), non-turners (bottom-right), spinners (top-right), and non-movers (top-left) from a birds-eye-perspective**. Excursion path is the first-person perspective movement along that trajectory.

One paradigm that shows striking behavioral differences between participants (and potential differences in underlying spatial updating processes, spatial representations and neural substrates) is a point-to-origin paradigm. Here, participants are presented with a visually simulated excursion path (Figure 1) consisting of a forward translation, a turn, and a second forward translation segment, after which they are asked to indicate the direction back to the origin of locomotion (Klatzky et al., 1998; Gramann et al., 2005; Riecke, 2008). As illustrated in Figure 2, for excursion paths including a left turn one would expect participants to point back into their left hemisphere. However, when only visual cues indicate self-motion, two response patterns emerged, based on whether participants point into the correct hemisphere or not, often termed “turner” and “non-turner,” respectively (Gramann et al., 2005; Riecke, 2008). That is, turner behavior is characterized by pointing overall to the correct hemisphere (e.g., participants pointing to their left for a 2-segment outbound path including one left turn). Thus, participants respond as if they succeeded in updating the presented heading changes, which could be associated with an correctly updated self-to-object (egocentric or 1st person perspective) centered reference frame (see Figure 2, bottom left). Non-turner response patterns, however, are characterized by pointing to the incorrect hemisphere (e.g., right for outbound paths including a left turn). Thus, participants respond as if they failed to update their heading and are still facing their original heading. This could be associated with an object-to-object (allocentric or 3rd person perspective) centered reference frame that does not change orientation, or a non-updated egocentric reference frame. The link between turner and non-turner response patterns with egocentric and allocentric reference frames is one possible interpretation of the response patterns of left-right hemisphere errors observed in about 20–100% of participants (see Table 1). Other potential explanations for left-right hemisphere errors in point-to-origin tasks include reference frame conflicts or sensorimotor interference between the instructed or cognitive heading (i.e., the updated reference frame) and participants' perceived or physical heading (which remained unchanged) as discussed in more detail below (Klatzky et al., 1998; Avraamides et al., 2004; Riecke, 2008, 2012). Note that such left-right hemisphere errors (non-turner response patterns) tend to disappear completely when participants actually perform the heading change during the outbound path by walking the outbound path or at least physically rotating (Klatzky et al., 1998; Avraamides et al., 2004). However, more recent studies suggest that physical rotations alone cannot reliable eliminate all non-turner behavior, indicated by some participants still showing left-right hemisphere errors despite full-body physical rotations (Sigurdarson et al., 2012; Sigurdarson, 2014).

**Table 1:** **An overview over turner studies, the used parameters and the percentage of turners ordered by the percentage of turners**.

**Study**	**Response mode**	***n***	**Context**		**Sensory information**						
			**Answer**	**Scene**	**Visual**	**Proprio-ceptive**	**Vesti-bular**	**Visual**	**Horizontal resolution**	**FOV**	**% of turners**
Klatzky et al., 1998	Blind walking	10	Point	Blind	No	Yes	Yes	Blind	0	0	100
Klatzky et al., 1998	HMD and Turn	10	Point	Starfield	Yes	No	Yes	HMD	800	44 × 33	100
Avraamides et al., 2004	Verbal	20	Describe	Blind	No	No	No	Blind	0	0	100
Riecke et al., 2012	Standard^a^	24	Point	Naturalistic	Yes	No	No	HMD	?	?	87.5
Sigurdarson et al., 2012	Real turn	12	Point	Naturalistic	Yes	No	No	HMD	800	32 × 24	83
Sigurdarson et al., 2012	Visual turn	12	Point	Naturalistic	Yes	No	Yes	HMD	800	32 × 24	83
Riecke, 2008	Standard	16	Point	Ground plane	Yes	No	No	Projector	1400	84 × 63	62
Riecke, 2008	Angle announced	24	Point	Ground plane	Yes	No	No	Projector	1400	84 × 63	54
Plank et al., 2010	Standard	37	Select	Tunnel	Yes	No	No	Projector	800	41 × 41	54
Gramann et al., 2010	Standard	12	Select	Tunnel	Yes	No	No	Projector	?	41	52
Gramann et al., 2012	Experiment 2	11	Select	Starfield	Yes	No	No	Monitor	?	47 × 35	50
Gramann et al., 2005	All conditions	43	Select	Tunnel	Yes	No	No	Monitor	?	?	47
Riecke and Wiener, 2007	Standard	20	Point	Plane	Yes	No	No	Projector	1400	84 × 63	45
Riecke, 2012	Standard	20	Point	Ground plane	Yes	No	No	Projector	1400	84 × 63	45
Goeke et al., 2013	Online	260	Select	Starfield	Yes	No	No	Monitor	1024	?	37
Chiu et al., 2012	Standard	20	Adjust	Tunnel	Yes	No	No	Projector	?	206	35
Goeke et al., 2015	Online	1148	Select	Starfield	Yes	No	No	Monitor	1024	?	~30
Kitson et al., 2015	Standard	39	Select	Starfield	Yes	No	No	Projector	Ranges	Ranges	28
Klatzky et al., 1998	Only HMD	10	Point	Starfield	Yes	No	No	HMD	?	44 × 33	0
Avraamides et al., 2004	Imagine and walk	20	Turn	blind/Real	Yes	No	No	Blind/Real	0	0	0

a *standard: point back to the origin of locomotion using a modified joystick or pointing device. ?, unknown*.

While previous studies focused predominantly on left-right hemisphere errors and the potential underlying turner and non-turner strategies, a recent study using visually-presented point-to-origin tasks described two additional response patterns based on anterior-posterior pointing errors, termed “Non-mover” and “Spinner” (Kitson et al., 2015; Riecke et al., 2016): On the one hand, Non-movers point in the correct left-right hemisphere (e.g., left for left turns), similar to turner response patterns, but instead of pointing posterior (as one might expect for correct pointing responses) they point anterior as illustrated in Figure 2 (top left). That is, non-movers respond as if they not only failed to update heading changes but also the movement itself, as if pointing from the origin to the end location. On the other hand, Spinners commit both left-right hemispheric and anterior-posterior errors at the same time. That is, they responded as if they arrived at the end point, turned 180 to face the path, and then pointed to the origin from the new orientation (see Figure 2, top right). Note that “Non-mover” and “Spinner” descriptions are currently only hypotheses about potential underlying mechanism (similar to the notion of turner and non-turner), and are an attempt to make sense of these unexpected answering patterns of anterior-posterior errors in Riecke et al. (2016) that has received little if any attention in previous studies.

Riecke did discuss several participants who consistently pointed in in the left-right reversed hemisphere and frontal, which cannot be simply explained by a failure to update one's heading (Riecke, 2008, 2012). However, their data would be incompatible with a spinner answering mode as described above. Instead, Riecke proposed that “there are some left-right inverters that apparently did update the visually displayed heading changes, but for some reason produced a left-right mirrored response, despite correctly perceiving the simulated turning direction. Post-experimental debriefing suggests that they might initially have been uncertain about the proper response and for whatever reason picked the wrong, left-right reversed, strategy and later continued to employ that same strategy.” (p. 170).

To explain the consistently observed hemisphere errors and non-turner response patterns, different researchers put forth different hypotheses about potential underlying factors and strategies that we will discuss more in the following. Note that we try here to consistently distinguish between observed behaviors (e.g., hemisphere errors termed “non-turner” response patterns) and potential underlying processes and representations, such as egocentric vs. allocentric representations or different strategies. To remain consistent with the literature, though, we keep using established terminology like “non-turner” when referring to left-right hemisphere errors, even though the term non-turner might suggest underlying process. (Gramann, 2013) argued that nonturners respond as if they had not turned and are still facing the original direction. They hypothesized that participants solve the task in a more abstract and disembodied way, by applying and allocentric reference frame that does not rotate during the passage. Thus, what might be thought to be an error in solving the task was hypothesized to be the result of a different strategy, where the answer was expressed in a different reference frame.

Avraamides et al., (2004) showed that an increased error (corresponding to non-turner behavior) did not arise when participants performed an imagined point-to-origin task and answered using spatial language instead of embodied pointing in the form of turning-to-face-the-origin. The researchers hypothesized that the non-turner answers in the pointing response mode are due to the strong attachment of the pointing gesture to the current perceived body orientation (“perceptual heading”), that is, aligned with the hypothetical egocentric eference frame of their original heading, which is in conflict with the cognitive or instructed heading.

Similarly, Riecke et al. (Riecke and Wiener, 2007; Riecke, 2008) argued that non-turner response patterns could be explained by sensory interference and reference frame conflicts between participants' unchanged physical orientation and the visually simulated or otherwise instructed orientation. According to the interference hypothesis, these conflicting perspectives can lead to interference at the response level especially when an embodied response mode like physical pointing or turning one's body to face the origin are used (Presson and Montello, 1994; May, 1996; Wraga, 2003; May, 2004; Wang, 2005; Riecke and McNamara, 2007).

Furthermore, Riecke proposed turner and non-turner behavior might be related to online spatial updating vs. more cognitively demanding offline computations (Riecke, 2012). That is, turners showed overall low response latencies and little systematic increase of response latencies with turning angles, which is compatible with online spatial updating to perform the point-to-origin task, similar to response patterns observed in prior spatial updating studies (Rieser, 1989; Presson and Montello, 1994; Farrell and Robertson, 1998; Riecke et al., 2007). However, non-turners showed considerably longer response latencies, which also systematically increased for larger turning angles. Riecke proposed that non-turner responses might thus be based on more effortful offline and after-the-fact computation of the desired homing direction. If the homing response was already computed during the outbound path, responses should be fast and little additional computational resources should be needed for larger turns (Riecke, 2012).

Note that the latter hypotheses are notably different from the one used by Gramann. While they agree that participants giving turner answers update their egocentric reference frame according to the given stimulus (i.e., imaginary walking, visual flow, etc.), they propose different explanations for the non-turner responses. Gramann explains non-turner behavior as a different strategy of solving the task using an allocentric reference frame. Avraamides and Riecke see non-turner answers as a result of the conflict between ones virtual, imagined, or instructed body orientation (cognitive heading) and ones actual, physical body orientation (perceptual heading). Here, non-turner answers are not considered valid answers in an allocentric reference frame but errors due to an overriding of the instructed (updated) egocentric reference frame with a physical egocentric reference frame of the original orientation. However, (Avraamides et al., 2004) found this conflict is largely reduced or removed when spatial language is used to give the answers, supposedly because of the more abstract and less embodied nature of spatial language compared to bodily pointing; this strategy might be closer to a more cognitive representation of heading.

To enable a neutral discussion of the phenomenon, in this study we will use the terms turner and non-turner as a shorthand for referring only to behavioral observation—whether participants point into the correct hemisphere and incorporate simulated or otherwise instructed heading changes into their response or not, without making an implicit assumption of which underlying reference frames or strategies they might use. Similarly, the terms spinner and non-mover are proposed as an easily memorable shorthand to describe response patterns based on anterior-posterior errors with or without left-right hemisphere errors, respectively.

In order to better understand why some participants tend to show turner vs. non-turner response patterns in virtual point-to-origin tasks, several studies investigated potentially contributing individual factors such as gender, video gaming experience, ethnicity, response mode, navigation skills, cardinal direction proficiency, and decision certainty (Avraamides et al., 2004; Riecke, 2008, 2012; Goeke et al., 2013, 2015; Gramann, 2013). Non-turner behavior has been shown to correlate with lower mental spatial abilities in some studies (Riecke, 2008) but not others (Riecke, 2012) although a trend seems common. Goeke et al. (2013) reported females to be more likely to show non-turner responses, confirming trends observed in prior studies (Riecke, 2008, 2012). Video gaming experience does not seem to correlate with non-turner behavior (Riecke, 2008, 2012; Goeke et al., 2013). Complicating the investigation of factors underlying non-turner behavior is the fact that non-turner rates vary widely between studies, as summarized in Table 1, with a multitude of methodological differences potentially contributing. In general, though, non-turner rates tend to be lower when rotations are physically performed and more naturalistic stimuli are used. In sum, individual factors determining response patterns and underlying strategy selection seem varied and interrelated, such that a coherent picture has yet to emerge. Most prior findings were also limited by relatively small sample sizes, leading to limited power in detecting potentially underlying individual factors. This motivated Goeke and colleagues to conduct online studies (Goeke et al., 2013, 2015), and motivated us to conduct experiments in a shared classroom setting where we could obtain both sufficiently large participant samples and ensure participants had a chance to ask questions in case instructions might be unclear or ambiguous, which can happen in spatial orientation experiments.

The first large cross-sectional study investigating the turner and non-turner phenomenon was an online study conducted by Goeke et al. (2013). Their sample contained (after preprocessing) 260 participants from 15 countries, with the majority from Spain and Germany. The task contained left or right (yaw) turns as well as up and down turns (pitch). Answers were given via selecting one of four 3D arrows. In their analysis they found females predominantly were non-turners whereas males were both non-turners and tuners with similar probabilities. Turners had higher cardinal direction proficiency and decision certainty compared to non-turners. This was not the case for self-estimated general navigation skills or video gaming experience, which revealed no difference between groups. In a similar study by Goeke et al. (2015), they investigated gender, age and cultural background for 1148 participants who completed an online navigation task. They did not find a significant difference for gender or age with respect to non-turner behavior. However, they did find that North Americans were more likely to be non-turners, Latin Americans more likely to be turners, and Europeans and Asians were in between though slightly leaning toward being non-turners over turners. Overall, it seems that a multitude of known and unknown factors influence whether participants show turner or non-turner responses, leading to often widely varying ratios of turners to non-turners in different studies. We will be using some of the same variables of previous studies in order to narrow the ratios or, if we find a completely different ratio than expected, examine why there is great variability across studies.

One potential influence on virtual navigation strategy is ethnicity. A large body of literature has well established the link between culture and cognitive style (Norenzayan et al., 2007; Varnum et al., 2008; Kitayama et al., 2009; Kitayama and Cohen, 2010). Western cultures, such as the United States, tend to exhibit a more independent and analytic social orientation: emphasizing uniqueness, having relatively low sensitivity to social cues, and encouraging behaviors that affirm autonomy. On the other hand, other cultures such as China tend to exhibit a more interdependent and holistic social orientation: emphasizing harmonious relations with others, promoting sensitivity to social cues, and encouraging behaviors that affirm relatedness to others (Kitayama and Cohen, 2010; Varnum et al., 2010). On the basis of such evidence, the link between social orientation and cognitive style has been widely accepted. Goeke et al. (2013) suggest cultural background as a possible underlying factor on reference frame proclivity. In Goeke et al. (2015), their online navigational study of 1823 participants found the majority of North-Americans showed non-turner behavior, while Latin-Americans predominately exhibited turner behavior. Europeans and Asians were in between these two response patterns. Together, this suggests a possible relation between cultural background and response patterns for virtual navigation tasks. To further investigate this, and because of the diversity of ethnic groups available at our location, we look at ethnicity as a possible factor in spatial response patterns and potential underlying strategies and reference frames.

Based on the prior research discussed above, the current study was designed to address three main goals:

1. Replicate the gender bias found in Riecke (2008) and Goeke et al. (2013). We hypothesize, based on the literature, females are more likely to be non-turners compared to males.
2. Extend the findings of Avraamides et al. (2004), predicting a higher amount of turners when spatial language is used instead of a more embodied response mode that might lead to larger sensorimotor or reference frame interference effects. To this end, we will use written spatial language vs. top-down pictograms as response modes.
3. Investigate a possible influence of ethnicity on turner vs. non-turner proclivities. The literature suggests eastern cultures will predominately exhibit non-turner response patterns while western cultures will lean toward turner response patterns, yet (Goeke et al., 2015) have found the opposite. It remains unclear how ethnicity affects answering patterns in virtual spatial navigation.

To answer these three questions, we designed our study with the idea of having a very large sample size to cope with intrinsically noisy strategy classification data and high individual differences. We collected data from Canada and Germany. We used a design that could be executed with many participants simultaneously, showing the stimulus on a projector and recording the answers via a paper questionnaire. This way, we were able to perform the experiment in lecture halls at the beginning of regular courses, and could ensure that participants could ask questions to help reduce potential misunderstandings of the task requirements or procedures. We chose a small number of trials, since earlier studies have shown that response patterns are relatively stable over time (Goeke et al., 2013). As a consequence of the study design, we could not directly employ the same answering modes as in Avraamides et al. (2004). Instead, we used pictograms (i.e., a top-down schematic of a person's head with an arrow pointing in the direction to the origin as illustrated in **Figure 5**) for the arguably more embodied version and written spatial language for the equivalent of description on spatial language. We hypothesize that pictograms will trigger a more embodied process than written language. We are aware that these answering modes are somewhat more abstract that the ones used by Avraamides and, thus, expect weaker effects.

## 2. Materials and methods

### 2.1. Participants

**Figure 3:**
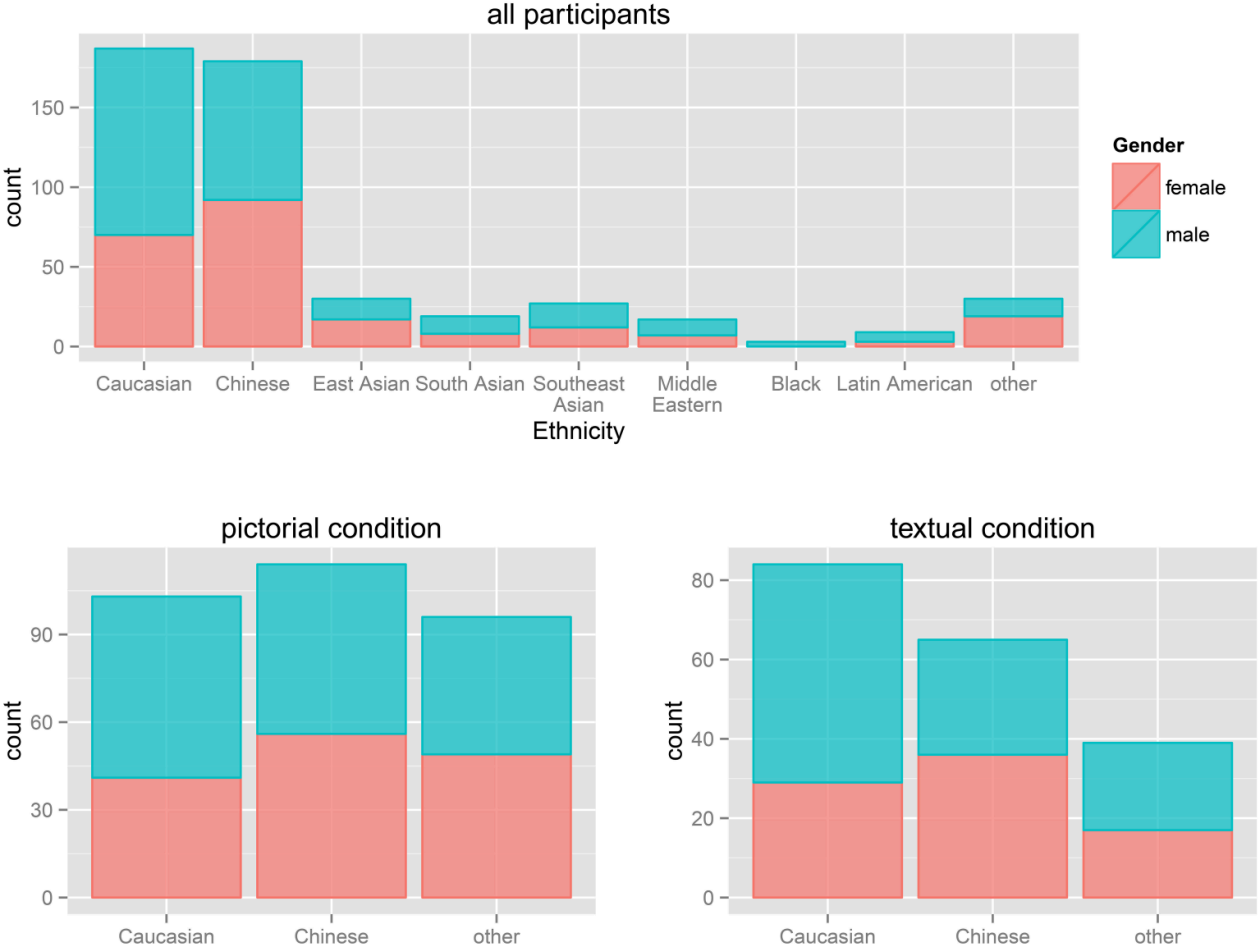
**Demographics of the participants**. The two main ethnicity groups were Caucasian and Chinese, all other Ethnicities were pooled into a third group. Two thirds of the Caucasian participants were male, and for all other groups the male female ratio was about one to one. This distribution is also reflected in the allocation to the two response modes (lower two plots).

A total of 507 participants took part in the study: 228 female, 273 male, and 6 NA. The average age was 20.5 years (*SD* = 3.2). We recruited a diverse spectrum of participants from 3 universities: Simon Fraser University (244 participants) and the University of British Columbia (183 participants) both in Greater Vancouver, Canada, and the University of Osnabrück in Germany (104 participants). An effort was made to recruit a sample with high ethnic diversity (see Figure 3). For a more detailed account of the number of participants in each subgroup, see Table 2. Participants were not reimbursed. This study was approved by the Simon Fraser University Office of Research Ethics (ORE).

**Table 2:** **Number of participants in each of the subgroups**.

	**Caucasian**	**Chinese**	**Other**						
			**East Asian**	**South Asian**	**Southeast Asian**	**Middle Eastern**	**Black**	**Latin American**	**Other**
Total	117, 70	87, 92	13, 17	11, 8	15, 12	10, 7	3, 0	6, 3	11, 19
Pictorial	62, 41	58, 56				47, 49			
Textual	55, 29	29, 36				22, 17			

*Numbers are listed as Male, Female. Seven subgroups (excluding Caucasian and Chinese) are grouped together under the heading “other” for pictorial and textual response modes*.

### 2.2. Stimulus and apparatus

**Figure 4:**
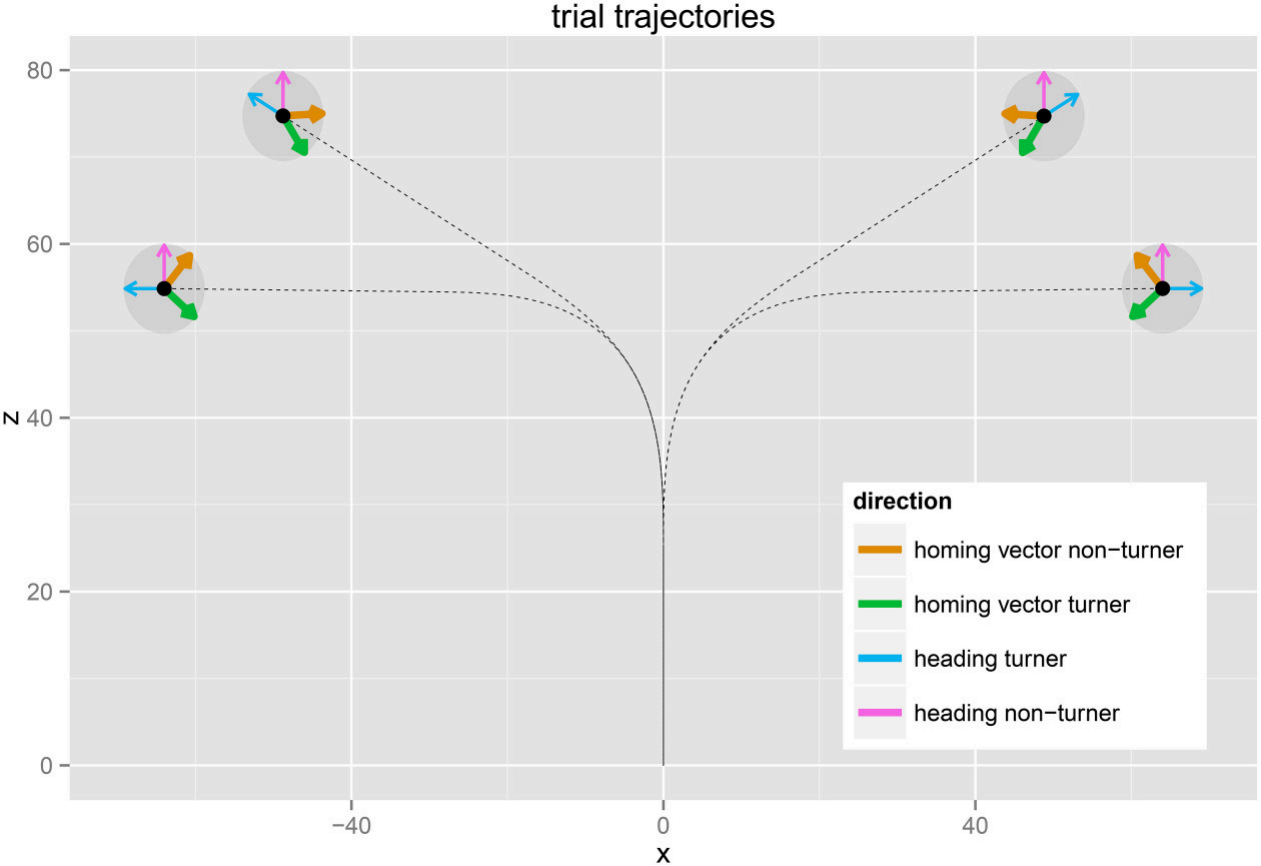
**The trajectories of the four trials from a birds-eye-view perspective**. Thin arrows indicate the heading at the end of the trajectory for turners (blue) and non-turners (purple), and the thick arrows depict the predicted homing directions for turners (green) and non-turners (brown). X and Z axes are the displacement along the ground plane in meters.

Participants were shown a passage through a virtual star field (see: http://player.vimeo.com/video/101479676? and Figure 1), providing optical flow without any landmarks. Participants were seated in medium to large sized lecture halls viewing the star field on the available projector and screen. Participants were grouped toward the front-center area of the lecture hall to avoid small field of views and provide roughly comparable viewing conditions. Trajectories consisted of an initial straight path, followed by a curve and a second straight path at the end. Curve angles used for the four trials were 60° *left*, 90° *right*, 90° *right*, and 60° *left*, respectfully (paths are illustrated in Figure 4). The velocity profile was smoothed to make the stimulus less artificial and prevent motion sickness. The first linear part included a 1*s* linear acceleration phase with $10\frac{m}{s^{2}}$, followed by a constant movement with $10\frac{m}{s}$ for 2*s*. The turn was divided into an angular accelerating half and a decelerating half, the constant acceleration being $15\frac{◦}{s^{2}}$, resulting in an overall turn time of 4*s* for 60° and 5*s* for 90°. The second linear part consisted of a 3*s* constant linear movement and 1*s* deceleration—slightly longer than the first part.

Velocities and distances are abstract in a star field environment and subjective perception highly depends on the star field parameters chosen (e.g., star size, area, and visibility range). Passages were programmed using *Worldviz Vizard 4.0*. Code for the study can be found online (http://github.com/leftbigtoe/starfield) and can be executed with the free trial version of *Vizard 4.0*.

**Figure 5:**
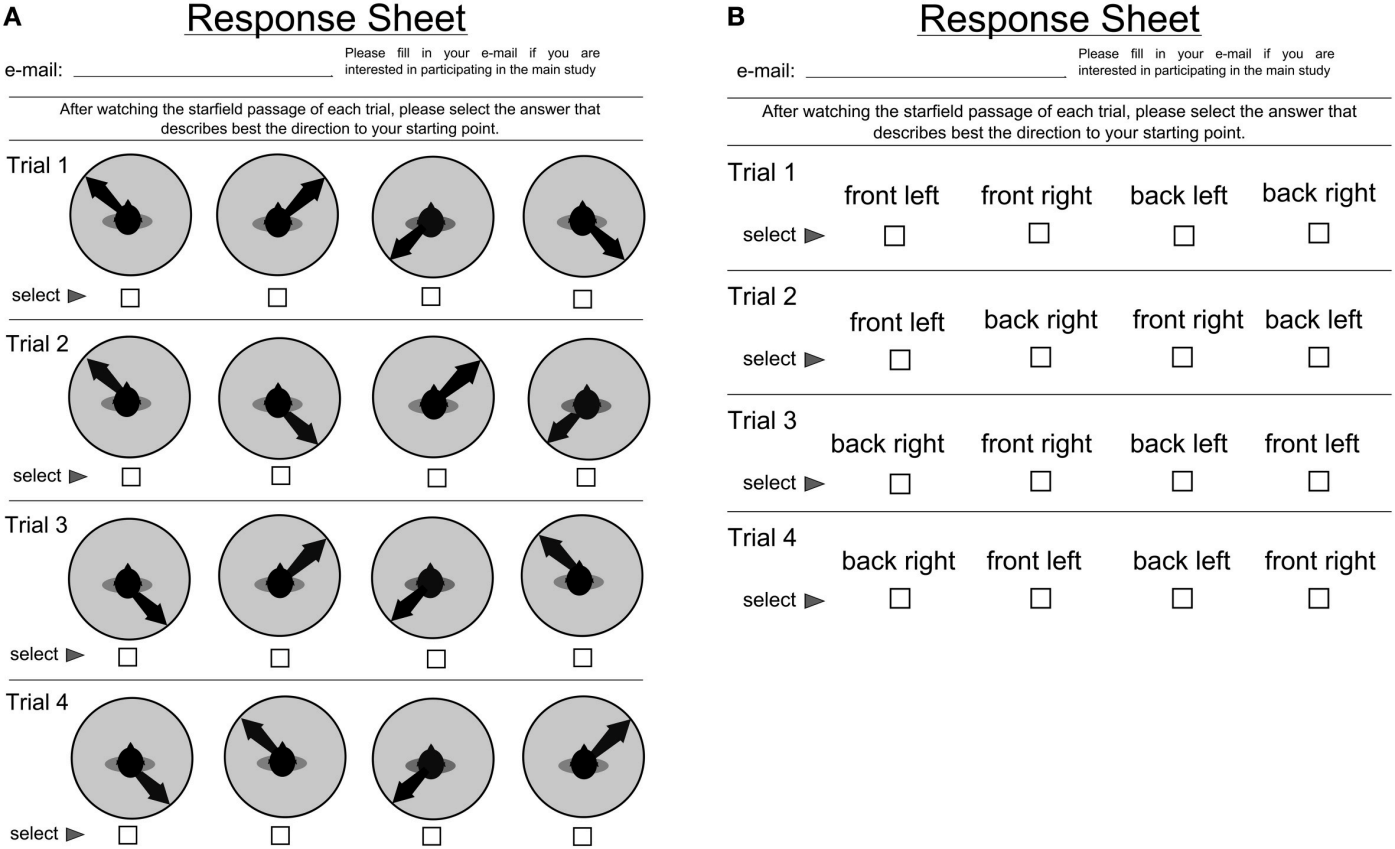
**(A)** Questionnaire for the pictorial response mode **(B)** Questionnaire for the text response mode.

Answers were given via a multiple choice questionnaire (see Figure 5). For each trial of the point-to-origin task, the same four possible answers could be selected: front left, front right, back left, and back right for both the textual response mode and the pictorial response mode. For each trial, the sequence of four possible responses was randomized to avoid answering tendencies. The response form was folded and sealed with tape, with the demographic information questionnaire inside to prevent possible task performance bias. The stimulus was shown on classroom projectors and lights were dimmed where possible. Participants were asked to group as closely as possible around the projector to minimize extreme viewing angles.

### 2.3. Procedure

The experiment took place either at the beginning or at the end of the classes. The lecturer introduced the experimenter, followed by distribution of informed consent forms. All students volunteering to participate signed the consent form and were randomly handed one of either a pictorial or text response form. The experimenter then explained the task until no participant had further questions. Participants were asked to select the answers as quickly and intuitively as possible. They were also asked not to copy from their neighbors or discuss their answers until after the experiment. Trials were shown to the class, pausing after each trial until everyone finished. No questions were answered that could provide feedback. After completing the task, the room was illuminated again and participants were asked to open their forms and fill out the demographics questionnaire. In total, the experiment took approximately 10 min.

### 2.4. Preprocessing

Before the analysis, preprocessing was performed on the collected data. Only participants who provided data for ethnicity and gender, and had no missing answers for the navigation task were used (*n* = 6 participants excluded). For each trial, strategy was classified (turner, non-turner, non-mover or spinner), in accordance with previous studies (e.g., Goeke et al., 2013), where participants were classified as users of the respective strategy based on consistent strategy use in 75% of the trials. All others were classified with no preference. Only three participants were classified as spinners. We excluded participants classified as spinners from further analysis due to sparseness of data (*n* = 3 participants excluded). Statistical analysis was performed with the remaining *n* = 498 participants. Given the 75% boundary and four trials per participant, we expect noise in the data, and predict online experiments might have similar or larger issues of noise because there is even fewer ways to check if participants are paying attention and understand the task. The current study and online studies should ideally be replicated in a lab setting to test for potential systematic influences of presentation type.

### 2.5. Data analysis

R 2.15.2 was used for data analysis. A multinomial regression model was used for statistical analysis and a likelihood ratio test of the parameters was done using an ANOVA.

## 3. Results

### 3.1. General response behavior

**Figure 6:**
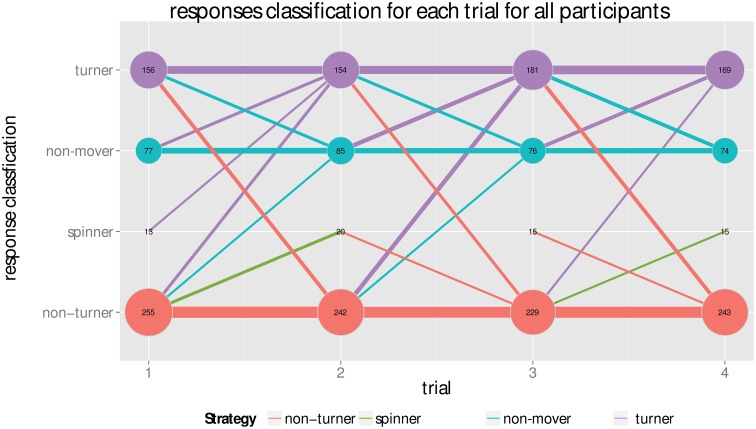
**Total counts of answering types per trial**. Y position and color of the dots indicate the type of the answer, x position the trial and area of the dot corresponds to the count, also given by the number within the dot. The bars indicate how many changed from giving one answer type in a previous trial to which answer type in the next trial, e.g., a bar from non-mover in trial 1 to turner indicates the amount of participants that changed from giving a non-mover response in the first trial to a turner answer in trial 2. Thickness again stands for amount of people changing in this way. A cutoff of *n*>5 for the bars was chosen to only show stable trends. Response classifications are relatively stable. The turner response draws the most participants over time from all other responses and is the only response that is growing overall while a spinner response is the most isolated. The interaction between non-mover is highest with the turner answers, giving more evidence that non-movers might be turners overestimating the turn. Non-turner interacts moderately, mainly with the turner answers and the spinner answers.

Total counts of responses over the trials (see Figure 6) show relatively stable response classifications, the two most prominent being non-turner answers (48.35%) and turner answers (32.93%). A smaller amount of participants gave non-mover or spinner responses, mainly non-mover, in the direction of the turn (15.57%). Very few spinner responses, in the opposing direction of the turn, were given (3.14%). Both turner and non-turner response patterns were expected—turner responses as they constitute the correct response, and non-turner responses as the were consistently reported in the literature and can be interpreted by failures to incorporate heading changes and responding from a non-updated reference frame. Non-mover and spinner responses, however, are not correct in either reference frame. Note that a frontal pointing in the direction of the turn (non-mover response pattern) could be explained in two possible ways. First, by a turner who overestimated the turn (i.e., over 135°). In this case, the starting point is in the frontal hemisphere. Second, participants could have misunderstood the task and pointed from the starting to the end point, or did so because it was the cognitively less demanding strategy; a few participants reported this after the experiment. For a frontal pointing in the opposite direction of the turn (spinners), one possible explanation is participants responded as if they completed the turn, turned to face the path they came, and then pointed to the origin. Another explanation is participants responded with a wrong answer due to inattentiveness or distraction, since spinner does not seem to be a very stable strategy: 36 participants (7.19%) gave a spinner answer once, 8 participants (1.6%) gave it more than once, and only 3 participants more than twice (0.6%).

**Figure 7:**
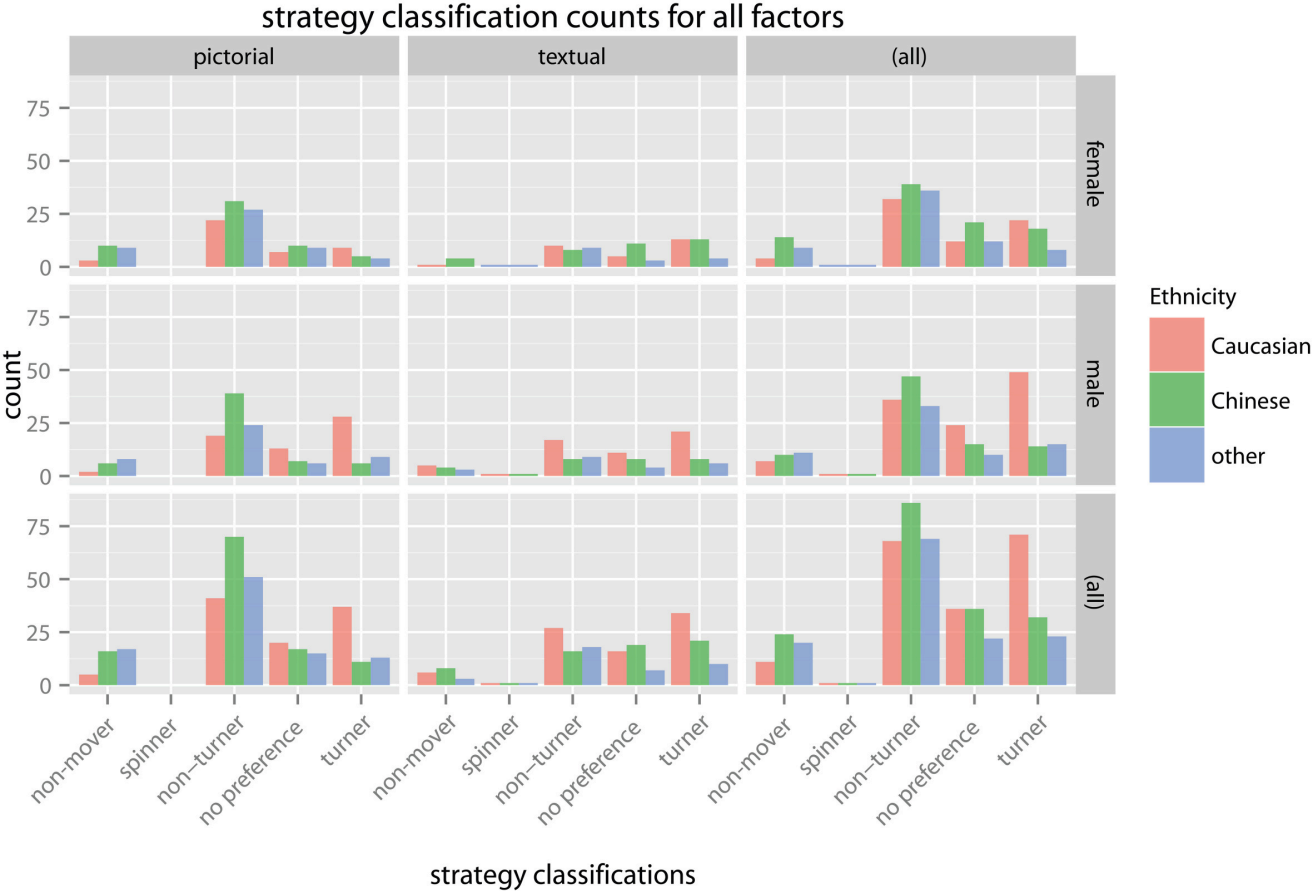
**Total counts of preferred response classifications factored out into each of the model factors response mode, ethnicity and gender and respective marginal sums**. It can be seen that the two most dominant classifications were turner and non-turner followed by no preference, while the frontal pointing classifications, especially spinner, were quite rare.

The overall counts of classification according to the 75% criterion (i.e., participants that used the same response mode in 75% of the trials) can be seen in Figure 7. As expected, the two most prominent classifications were non-turner (44.78%) and turner (25.3%). 11.04% were classified as non-mover users and only 0.6% had spinner as their preferred response mode. 18.88% of the participants did not show a clear preferred response mode and were classified with no preference. Evident in this overview is the high amount of non-turners in the pictorial response mode (51.8%) compared to the text response mode (33.0%), and the high amount of male Caucasian turners (42.2%), especially in the pictorial response mode (45.2%).

### 3.2. Multinomial regression model

**Figure 8:**
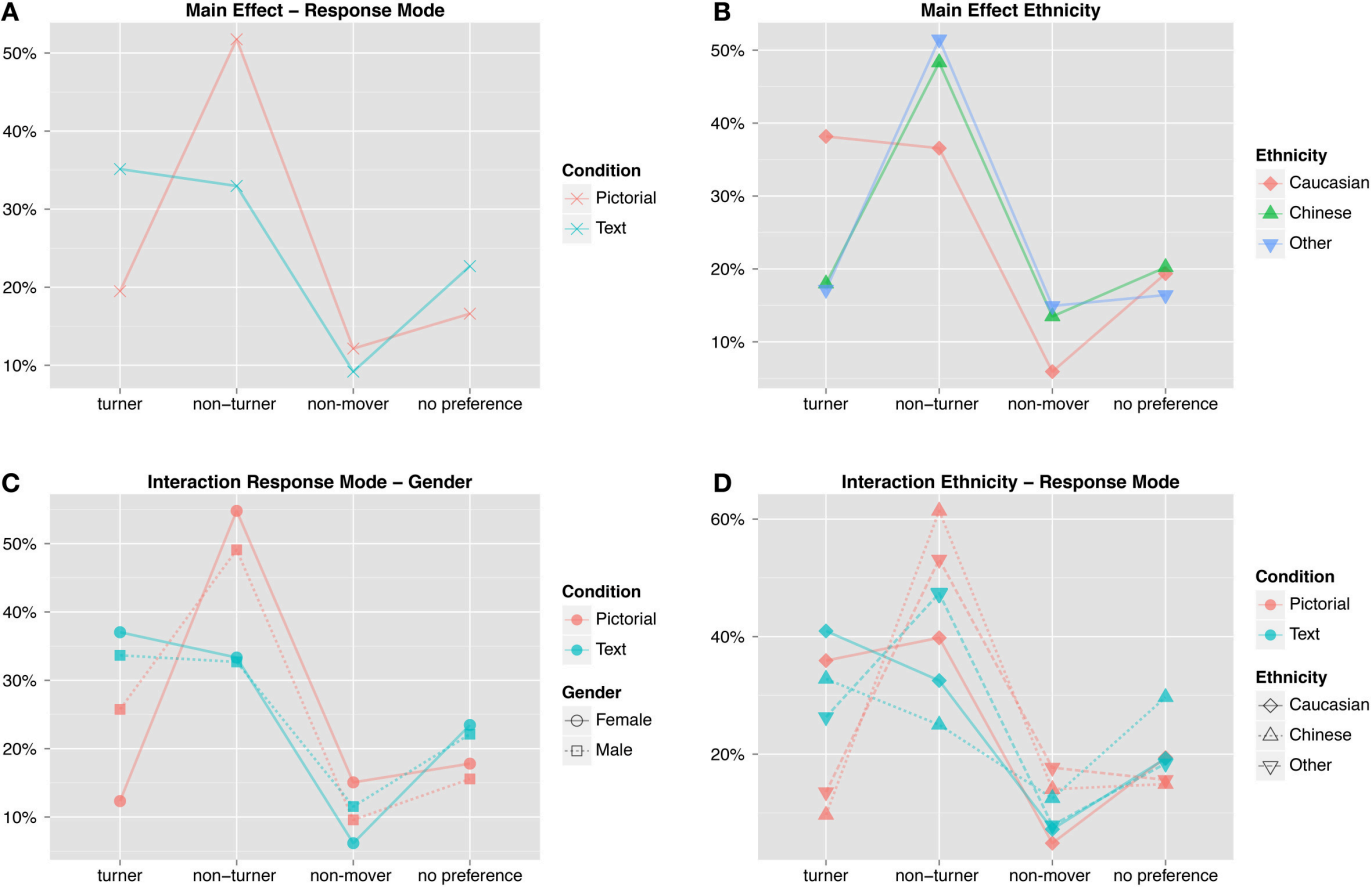
**Percentages of preferred response classifications illustrating the significant main effects and interactions**. **(A)** Participants in the pictorial condition preferred a non-turner response classification, whereas those in the text condition preferred either a turner or non-turner response mode. **(B)** Caucasians have a much greater preference for a turner response mode than either Chinese or Other ethnicities, who prefer a non-turner response mode. **(C)** Males have a greater percentage of turners than females in the pictorial condition. **(D)** There is a high percentage of Caucasian turners in the pictorial condition, compared to Chinese and Other ethnicities. There is also a difference in Chinese non-turners between the pictorial and text conditions.

For statistical analysis a multinomial regression model was fitted. We included the factors response mode, ethnicity, gender, and all interaction terms to model the preferred response mode. Accuracy of the model on the training data was 49.0% compared to 25% chance level. The precise parameter values can be found in Table 2, with the significant effects summarized as percentage plots in Figure 8 and described below.

Likelihood ratio tests on the regression parameters revealed two parameters were highly significant: response mode ($p_{chi^{2}}<0.001$) and ethnicity ($p_{chi^{2}}<0.001$). The respective participant classification for response mode and ethnicity are summarized in Figures 8A,B. Further, the interaction terms ethnicity and response mode and response mode and gender were found to be mildly significant ($p_{chi^{2}}<0.05$), see Figures 8C,D. In contrast to earlier studies (Goeke et al., 2013), gender was not found to be significant at all. For an overview see Table 3.

**Table 3:** **Parameter values and standard errors of all parameters of and each respective outcome compared to the strategy baseline no preference**.

**Parameter**	**Non-turner**		**Turner**		**Non-mover**	
	**Estimate**	***SE***	**Estimate**	***SE***	**Estimate**	***SE***
(Intercept)	1.15	0.434	0.251	0.504	−0.847	0.69
EthnicityChinese	−0.0136	0.566	−0.944	0.744	0.847	0.822
EthnicityOther	−0.0469	0.58	−1.06	0.784	0.847	0.836
Response ModeText	−0.452	0.699	0.704	0.729	−0.763	1.29
GenderMale	−0.766	0.564	0.516	0.605	−1.02	1.03
EthnicityChinese:ResponseModeText	−0.998	0.915	0.156	0.999	−0.249	1.49
EthnicityOther:ReponseModeText	0.453	1.04	0.396	1.21	−12	0.66
EthnicityChinese:GenderMale	1.35	0.787	0.0226	0.988	0.87	1.25
EthnicityOther:GenderMale	1.05	0.821	0.701	1	1.31	1.25
ResponseModeText:GenderMale	0.508	0.876	−0.824	0.884	1.85	1.6
EthnicityChinese:ResponseModeText:GenderMale	−0.775	1.24	0.119	1.35	−1.37	1.94
EthnicityOther:ResponseModeText:GenderMale	−1.08	1.39	−0.276	1.56	10.4	0.66

Because of the small sample size in each subgroup we additionally ran a binary logistic regression, concentrating only on the main two groups Turner and Non-Turner. The results were similar to the multinomial approach we used. Likelihood ratio tests on the regression parameters revealed two parameters were highly significant (Table 4): response mode ($p_{chi^{2}}<0.001$) and ethnicity ($p_{chi^{2}}<0.001$); the interaction terms ethnicity & response mode were found to be mildly significant ($p_{chi^{2}}<0.05$); gender was also not found to be significant.

**Table 4:** **Model parameters of the multinomial regression models**.

**Parameter**	**LR *chi*^2^**	***df***	**$p_{chi^{2}}$**	
Ethnicity	26.8880	6	0.00015	^***^
Response mode	17.9785	3	0.00044	^***^
Gender	2.1589	3	0.54009	
Ethnicity:Response mode	14.3335	6	0.02612	^*^
Ethnicity:Gender	5.9970	6	0.42353	
Response mode:Gender	7.9853	3	0.04632	^*^
Ethnicity:Response mode:Gender	2.8220	6	0.83084	

*** 0.01;

* *0.05*.

### 3.3. Bootstrap confidence intervals for model performance

To further judge accuracy, a bootstrap analysis was conducted. For a review on bootstrap methods see Efron and Tibshirani (1986). Two kinds of bootstrap models were created: a naive one creating random classifications for every participant with uniform probability and one where the probability of the classifications were weighted based on the observed response classification counts. 10, 000 random classifications were created for each model and the confidence intervals calculated. The accuracy of our model lay outside of both bootstrap confidence intervals (naive: 23.5–28.7%, weighted: 29.7–35%) indicating a decent fit. A further observation is the model only made two classifications, non-turner or turner, but never non-mover or no preference. This inability of the model to discriminate between all four response classifications and the emergence of turner and non-turner as main response classifications indicates some correlation between some of the response classifications. No preference and non-mover both seem to be correlated to one of the main response classifications instead of being independent response classifications. However, the fact that there is more training data for the turner and non-turner classifications could possibly account for some of the bias of the model.

### 3.4. Main effects

**Figure 9:**
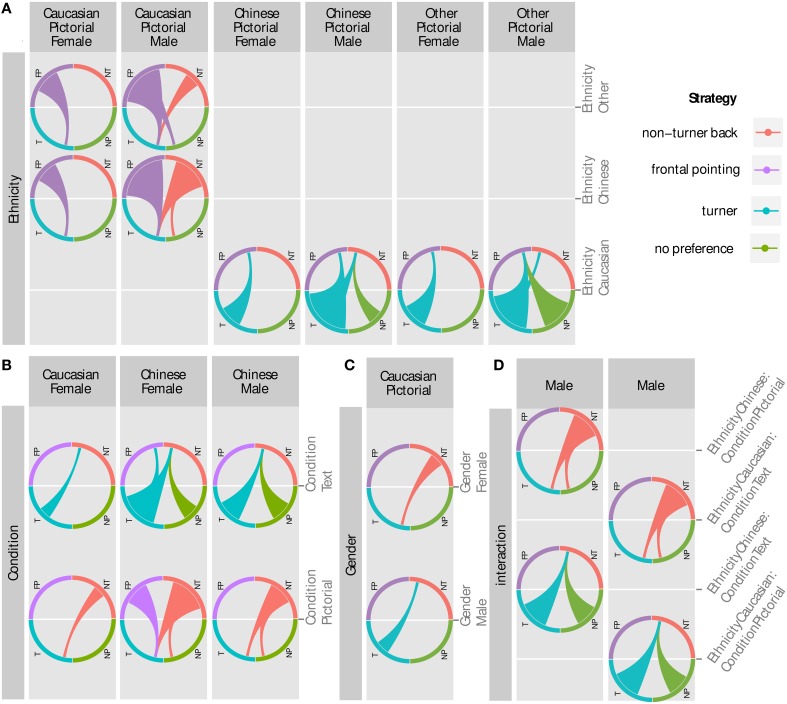
**Significant and reasonable odd ratios**. Each chord marks a significant comparison. The thin end is the baseline strategy, the thick end of the response that is more likely instead of the baseline. Example left circle of **(C)**: for Caucasians in the pictorial response mode being male means a classification as turner is significantly more likely than being a non-turner compared to being female (3.6 times more likely). **(A)** The effect of response mode was significant for female Caucasians and both genders among Chinese participants. They were more likely to be non-turners or frontal pointers (spinners and non-movers) in the pictorial response mode and turners or have no preference in the text response mode. **(B)** Gender related ORs were only significant for Caucasians in the pictorial response mode. Males were more likely to be turners while females were more likely to be non-turners. **(C)** All effects for Ethnicity only emerged in comparison to a pictorial baseline. Here Chinese and Other were more likely to be frontal pointers (men and women) or non-turners (only males). Vice versa, Caucasians were more likely to be turners compared to Chinese and Other, while having no preference was also more likely but only for males. **(D)** The interaction terms go into a similar direction than before, showing an opposing trend: while Caucasians are turners or have no preference in the pictorial response mode where Chinese are more likely to be non-turners, this reverses for both ethnicities in the text response mode. Here the effects only appear compared to a male baseline.

Before presenting and discussing the detailed odd ratios we will use the percentage plots in Figure 8 to provide a quick overview of the influence of all model parameters that were found to be significant. For a more detailed analysis of all odd ratios see Section 3.6 and Figure 9.

#### 3.4.1. Response mode

For the response mode, we observed an approximately equal split of turners and non-turners within the text condition (35.1 and 33.0%, Figure 8A). This is in stark contrast to the the pictorial answering condition where we see 51.8% non-turners and only 19.5% turners.

#### 3.4.2. Ethnicity

While the percentage of turners and non-turners within the Caucasian group is roughly equal (38.2 and 36.6%, see Figure 8B), there are far more non-turners in the Chinese and Other Ethnicities groups, which showed similar responses overall. For the Chinese group, we observed 48.3% of non-turners compared to 18.0% of turners and similarly 51.5% of non-turners and 17.2% of turners for Other Ethnicities.

### 3.5. Interaction effects

#### 3.5.1. Response mode—gender

There is an interesting interaction that becomes apparent when looking at the response classification per response mode split by gender (Figure 8C). In the pictorial condition, the response classification is more equally split for male subjects (25.7% turners, 49.1% non-turners) compared to female subjects (12.3, 54.8%). This is not the case for the text condition or the other response classifications.

#### 3.5.2. Ethnicity—response mode

The split between turner and non-turner answers is fairly equal amongst Caucasians for both response modes (pictorial: 35.9–39.8%, text: 41.0–32.5%), as illustrated in Figure 8D. This is, however, not the case for the other ethnicity groups. We observed a strong imbalance for Other Ethnicities within the pictorial condition (13.5–53.1%) while the distribution is closer together in the text condition (26.3–47.3%). In other words, while the Other Ethnicities group is approaching the answer distribution of the Caucasian group in the text condition the contrast is even more pronounced in the pictorial condition. The Chinese group shows a similar effect for the turner answers: while there are very few turners in the pictorial condition (9.6%) in this group there is a fair amount of turner answers in the text condition (32.8%). In contrast to the Other Ethnicities, however, there is a huge difference of non-turner response classification users between the pictorial (61.4%) and text condition (25.0%). Further, the Chinese group also has a large percentage of no preference answers in the text condition (29.7%) compared to the pictorial condition (14.9%), while this discrepancy is not present in the other groups.

### 3.6. Odd ratios

From the regression parameters of the multinomial regression model, we directly calculated the odd ratios (ORs) for more detailed interpretation of the results. An odds ratio (OR) is a measure of association between a property and an outcome. The OR represents the odds that an outcome will occur given a particular property, compared to the odds of the outcome occurring in the absence of that property. They are calculated by dividing the number of occurrences that a participant has *a* given *b* (the odds of *a* given *b*) divided by the number of occurrences of *a* given not *b*. An OR >1 shows a positive correlation of *a* with *b* while an OR < 1 indicates a negative correlation. ORs = 1 means no correlation.

In multinomial regression models, parameters with more than two factors are dummy coded as dichotomous variables and comparisons are always performed by using one of two possible values for a factor as baseline and comparing it against the other value. To capture all effects, a model was created for every possible combination of base cases and all significant odd ratios were extracted (Wald confidence intervals that did not contain 1). Note that changing the baseline values does not change the overall performance of the model, rather, it “phrases the result in a different way.” Due to the dichotomous dummy coding there is also a mirror symmetry among the reported effects (e.g., OR text makes turner instead of non-turner more likely and OR pictorial makes non-turner instead of turner more likely). This symmetry is also nicely visible in the plots (see Figure 9). We reported both ways to avoid introducing a bias by leaving too much implicit. In the next step, all odd ratios with values under 0.001 and over 100 were excluded. Those ORs were highly likely to be artifacts of sparse data, having huge confidence intervals, indicating their unreliability. Following, only ORs greater than one will be shown. Due to the dichotomous dummy coding of parameters, every effect indicating *x* to be less likely for a certain parameter having value *b* also means *x* is more likely if that parameter has its other possible value *a*. To avoid redundancy, we will only present ORs greater than one. ORs are plotted in Figure 9.

#### 3.6.1. Ethnicity (see Figure 9A)

All Ethnicity effects were found with the pictorial response mode as baseline. Chinese and Other Ethnicities were more likely to be non-movers instead of turners, compared to male Caucasians (Chin. OR: 14, Other E. OR: 12.45) and female Caucasians (Chin. OR: 6, Other Ethnicity OR: 6.75). Further, compared to male Caucasians, Other Ethnicities were more likely to be non-turners instead of turners (OR: 3.93). Chinese males were non-turners instead of no preference (OR: 3.81) or turners (OR: 9.58). Vice versa, Caucasians were more likely to be turners instead of non-movers, compared to Chinese (male OR: 13.99, female: 6) or Other Ethnicities (male OR: 12.45, female OR: 6.75). Male Caucasians were also more likely to have no preference (OR: 3.81) or to be turners (OR: 9.58) instead of non-turners, compared to Chinese. Lastly, male Caucasians were more likely to be turners instead of non-turners (OR: 3.93) or have no preference instead of being non-movers (OR: 8.67), compared to males of Other Ethnicities.

The effects of ethnicity again seem to be more pronounced when a male baseline is used, possibly explained by the extreme amount of male Caucasian turners. Another noteworthy observation is no significant difference between Chinese and Other Ethnicities, and their comparisons against Caucasians are quite similar. This can be interpreted in two ways: either a high similarity between the Chinese and Other Ethnicities or Caucasians are quite unusual in their navigation behavior compared to other ethnicities. It seems unlikely that the differences might be mediated by a difference in video gaming or navigation skills, since both were not significantly different in both groups, as revealed by a Kruskal Wallis Test (self rated navigation skills *H* = 0.17, *df* = 1, *p* = 0.68 and gaming *H* = 0.82, *df* = 1, *p* = 0.37). Compared to Goeke et al. (2015), our effects of cultural background on spatial reference frame proclivity were similar for Eastern cultures and different for Western cultures. Asians were primarily non-turners in both our study and Goeke et al. (2015). However, results are the opposite for Caucasians (European and North American in Goeke et al., 2015) found Caucasians to be primarily non-turners, while we found Caucasians to be primarily turners. Our results seem to vary along the dimensions discussed in cross-cultural psychology (i.e., Western vs. Eastern populations; Norenzayan et al., 2007; Varnum et al., 2008; Kitayama et al., 2009; Kitayama and Cohen, 2010). Here, Westerners are predicted to use a turner response pattern, and Easterners are predicted to use a non-turner response pattern. One difference between our study and Goeke's is in classification parameters along the cultural domain. Many of our participants were recruited in North America, so one might expect them to have similar cultural context, environment, and language. However, our participant pool is based in Greater Vancouver, Canada, an area that is highly multicultural. Many of our participants have multicultural backgrounds, and have language, upbringings and cultural influences that are not typical to North America. Therefore, we grouped by ethnicity given the multicultural backgrounds of our participants. Future studies will gather more participants from other countries, who have not moved to North America, in order to see if there are any differences from our present study.

#### 3.6.2. Response mode (see Figure 9B)

A significant effect of the response mode for Caucasians can only be observed among females (OR: 3.18), and a significant effect is present for both sexes among Chinese participants (male: 6.5, female: 10.07). In both cases, the pictorial response mode makes a non-turner response classification more likely compared to a turner classification. For Chinese participants, a non-turner response classification is also more likely compared to no preference (male OR: 5.57, female OR: 4.26). Among female Chinese, a non-mover classification also becomes more likely (OR: 6.5). On the other hand, the text response mode has the opposite effect, rendering turners more likely in the same groups: Chinese males and females are now turners instead of non-turners (male OR: 6.5, female OR: 10.8) and have no preference instead of non-turner (male OR: 5.57, female OR: 4.26). Chinese females were also more likely to be turners instead of non-movers (OR: 6.5). Among Other Ethnicities, no significant effects for response mode emerged. Effects are stronger compared to a non-turner response classification as baseline.

We replicated the results of Avraamides et al. (2004), showing that the use of spatial language indeed makes turner responses more likely. Moreover, we extended the findings, showing the effect also remains present for simple multiple choice response sheets using more abstract pictograms and written spatial language for indicating the direction of origin. Interestingly, this effect is not significant in male Caucasians, which could be due to an already quite high amount of turners in this group in the pictorial response mode. There was no effect within Other Ethnicities, which may be due to the heterogeneous composition of different ethnicities within this group averaging out any effects.

#### 3.6.3. Gender (see Figure 9C)

Gender effects only emerged among the Caucasians with the pictorial response mode as baseline. Here, males were more likely to be classified as turners (OR: 3.6) and females tended more toward being a non-turners (OR: 3.6). Gender also showed interaction effects with other factors, such as the stronger difference between male Caucasians and male Chinese participants compared to their female counterparts.

Against our expectations, females were not, in general, more likely to be non-turners than males, contradicting the results of Goeke et al. (2013). Gender was not found to be a significant model parameter, and it only turned out to be significant within the interaction term of the model. Examining further, we found that the only significant OR for gender was found in comparison to the Caucasian and Pictorial baseline. All in all, our results suggest that the gender effect found in Goeke et al. (2013), where most participants were from Germany and Spain, could be an artifact of a very specific task and sample instead of a general bias in reference frame use. However, our results are more parallel to that found in Goeke et al. (2015), where they did not find an overall gender effect.

#### 3.6.4. Interactions (see Figure 9D)

Only the interaction between Ethnicity and Response Mode yielded some significant ORs. The interaction again emphasized effects already seen before: in the pictorial response mode Caucasians are more likely to be turners (OR: 7.75) or have no preference (OR: 5.89), both compared to being a non-turner. The same holds for Chinese in the text response mode where they are also more likely to be turners (OR: 7.75) or have no preference (OR: 5.89). Consequently, male Caucasians are more likely to be non-turners in the text response mode (OR against no pref.: 5.89, OR against turner: 7.75) while the higher likelihood of a non-turner classification for Chinese males was found for the pictorial response mode (same ORs). The interaction effects show common directions instead of influences of single parameters for given baselines. Chinese and text push in the same direction as Caucasian and Pictorial, toward a turner or no preference classification, while Chinese and Pictorial and Caucasian and Text push in the other direction toward a non-turner classification. Comparing our study with (Goeke et al., 2015), the culture-gender interaction was the same. Caucasian males tended to be more turners than Caucasian females, and Asian males tended to be more non-turners than Asian females. Surprisingly, our results for ethnicity (no interactions) differed from Goeke et al. (2015) given that both studies used short videos of virtual passages through a star field and participants responded by selecting the direction pointing back to the starting position. In our study, male Caucasians were highly likely to be turners were as all other groups and subgroups were likely to be non-turners. The number of males in Goeke et al. (2015) was similarly high, so it appears the gender-ethnicity interaction does not account for the difference in our results. One possible explanation is the response mode. Goeke et al. (2015) used pictorial arrows pointing up, down, left, and right, while we used pictorial arrows pointing front-left, front-right, back-left, and back-right as well as the corresponding textual response mode. It is possible the different response modes used in these two comparable studies accounts for the differences in the Caucasian spatial reference frame proclivities, given our results showed response mode is highly significant. However, further examination into the ethnicity and response mode interaction is required. Moreover, the pairwise comparisons only show a significant difference for Caucasians, suggesting the interaction is strongly driven by the Caucasian subgroup, and implies different subgroups differed in variance or had an uneven amount of observations.

**Figure 10:**
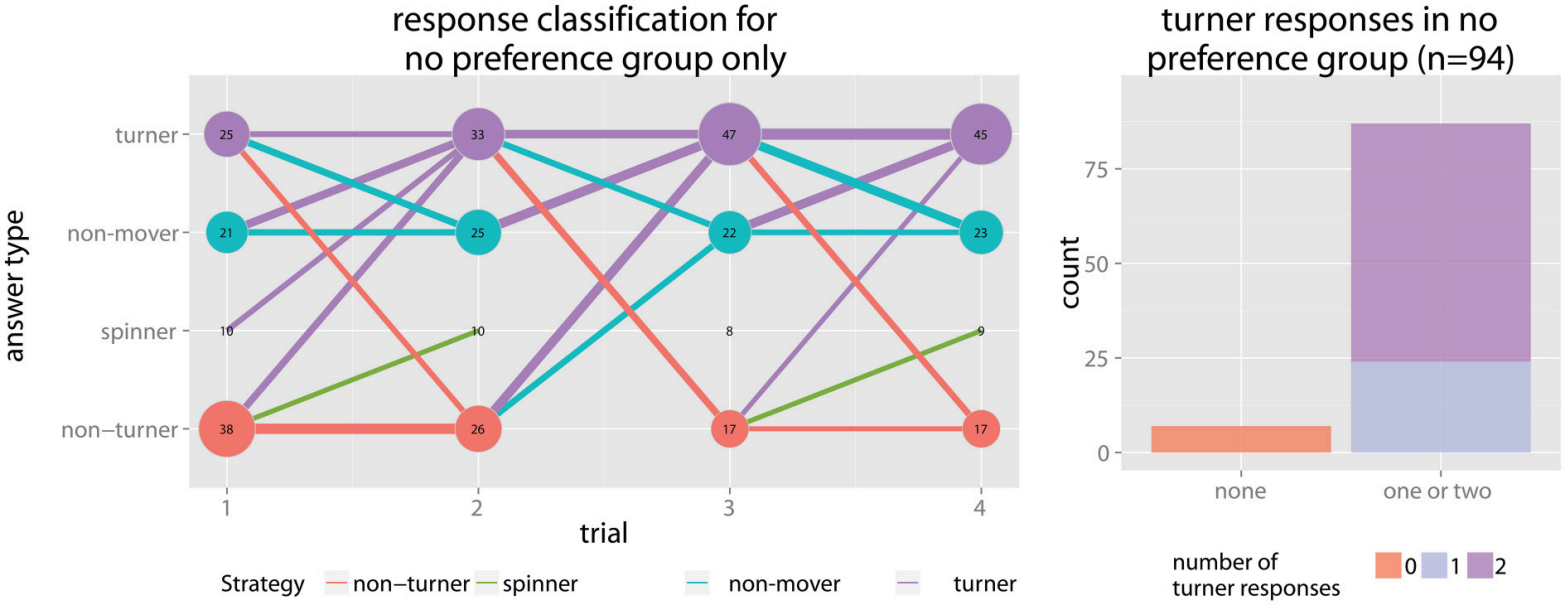
**(A)** Response mode graph for the no preference group. While the number of frontal pointing answers (i.e., non-movers and spinners) stay almost constant, the number of turner answers constantly grows and the number of non-turner answers shrinks. Also participants giving all sorts of answers before change to a turner answer in subsequent trials, while exchange among other answering types is more limited. **(B)** 87 participants (93%) within the no preference group gave at least one turner answer.

Another interesting observation is the effects grouping in a way where two response classifications are likely to appear together, with turner and no preference on the one side and non-turner and non-mover on the other. This connects to the emergence of turner and non-turner as main classifications of the model and its inability to make non-mover or no preference classifications. Although the two correlating classifications do not always appear together, they never appear in different combinations. This fact was also reflected by the classification behavior of the model that classified data into turner or non-turner but never into no preference or non-mover. While 93% in the no preference group gave at least one turner answer, this was only the case for 31% in the non-mover group. A possible explanation for the link between the turner and no preference response classifications could be that no preference acts as a kind of pre-stage to a complete turner response pattern. Participants with strong proclivities for the use of a non-turner pattern might start to give turner answers for some of the trials. The data even suggest a temporal development in which turner responses become more frequent among participants in the no preference group, as can be seen in Figure 10. The number of turner answers is the only one constantly growing and ends up being the most frequent question in the fourth trial. However, since the experiment only included four trials, conclusions about temporal development have to be taken with a grain of salt.

## 4. Discussion

### 4.1. Limitations

The small number of trials, especially the finding about a trend toward turner answers within the group classified with no preference, has to be taken with care. Because the study was conducted in classrooms, several limitations are present: a biased perception of the stimulus due to extreme viewing angle, interaction and copying between participants, and simple issues like lack of motivation or inattentiveness. Also, although the experimenter took care to explain the task thoroughly and participants could ask questions before the experiment started, the occurrence of non-mover responses suggests that those participants might not have perfectly understood the task. However, a similar argument could be made about non-turner responses. It might just be that participants resort to a simpler and/or cognitively less demanding response pattern and underlying strategies and required mental spatial transformations (non-mover or non-turner as compared to turner) when faced with a subjectively challenging spatial orientation task. Indeed, participants in virtual point-to-origin tasks often report the task as being “surprisingly extremely difficult” and give high ratings of perceived task difficulty, and (Riecke, 2008) observed that “not a single participant reported having any kind of natural or intuitive spatial orientation during the VR experiments, not even the VR-experienced lab members” (p. 167). Although we cannot rule out that some participants might have misunderstood the task, we minimized those issues wherever possible by pilot testing the instructions, giving participants a chance to ask questions before the experiment, and reducing potential systematic effects of remaining noise with a large sample size. In future studies, it could be useful to include a catch response that would help identify any lack of attention. Many participants expressed interest in knowing their own strategies at the end of the study, and most did not know whether they used a turner or non-turner response pattern. Future studies will further debrief participants, giving them a chance to discover their own strategies and elucidate underlying factors. A more in-depth mixed-method follow-up study could also be performed so the experimenter can see if the participant was confused about the task or made any errors.

### 4.2. Revisiting the hypothesis

Concerning the initial hypothesis of the study we can conclude the following:

#### 4.2.1. Gender effects are quite limited

Our results contribute to the controversy around gender differences in spatial navigation. We could not replicate a general influence of gender as in Goeke et al. (2013), but we could replicate (Goeke et al., 2015)'s later study incorporating culture background. A gender influence appeared only in the pictorial response mode and, even more interesting, only among Caucasians. This may be due to the extremely high amount of turners among male Caucasians. Sex differences in human spatial abilities are well established in the literature (Linn and Petersen, 1985; Voyer et al., 1995), the most stable difference being found for mental rotation tasks. Here, women scored significantly worse compared to men, which was assumed to be correlated with the female bias toward the use of landmark based strategies compared to orientation based navigation strategies (Astur et al., 1998; Dabbs et al., 1998; Moffat et al., 1998). However, this view was somewhat challenged by Parsons et al. (Parsons, 2004), who found that gender difference observed in mental rotation tasks vanished when a 3D virtual environment instead of a paper and pencil test was used for the task. They offered the possible explanation that it was the creation of a 3D representation from 2D drawings that actually caused or inflated the bias, not necessarily the task itself. If female participants in our study had higher difficulties in relating the 2D pictogram to the solution of the task, this could be an explanation for more female non-turners and for why this bias vanished in the text response mode. Moreover, our findings might offer a possible answer for the high controversy of gender differences in earlier studies. Our results can be read in the way that those differences are not universally present gender differences, but gender differences tied to cultural background, explaining why their presence or absence is highly dependent on the sample demographics.

#### 4.2.2. It is important how the question is posed

We were able to replicate the overall findings of Avraamides et al. (2004) (i.e., more turner responses for spatial language response mode) and extend them insofar as they also hold for a more abstract level were written spatial language and pictograms are used for answering instead of pointing and verbally responding with spatial language. Our results add more evidence to the hypothesis that non-turner answers may be due to a reference frame conflict or sensorimotor interference between one's mental or to-be-imagined orientation and current perceived body orientation that is more severe when answering mode is more embodied (Presson and Montello, 1994; Avraamides et al., 2004; May, 2004; Wang, 2005; Riecke and McNamara, 2007; Riecke, 2008). The pictogram response mode seems to have elicited similar reference frame conflicts as the actual physical pointing or turn-to-face origin ones in the Avraamides et al. (2004) and Klatzky et al. (1998) studies.

#### 4.2.3. Male caucasians appear to be a specific subpopulation

Caucasians, especially males, seem to be a specific subpopulation in our study when it comes to virtual point-to-origin tasks. The number of male Caucasians giving turner responses in the pictorial response mode was extremely high while in all other groups the trend was exactly the other way around, strongly in favor for non-turner responses. This effect might have carried over to several other effects: gender effect was only observed among Caucasians, response mode effect was not present for male Caucasians, and interaction effects were only present against a male baseline and in comparing Chinese and Caucasians. We currently have no conclusive possible explanation for this effect and further research is needed on this topic.

### 4.3. Further effects

An effect not hypothesized beforehand is the co-occurrence of front pointing (non-movers and spinners) with non-turner responses, and no preference responses. We concluded that the border between the main strategies non-turner and turner might be harder to draw than previously assumed, especially during the first trials of a point-to-origin task (see also discussion in Sigurdarson, 2014). Interestingly the trend in the no preference group went clearly toward turner responses. Along the lines of Avraamides' and Riecke's hypotheses, this could mean that some participants, after an initial confusion or uncertainty due to the reference frame conflict between actual and virtual/imagined body orientation, get to a point were they resolve the conflict and adapt the virtual orientation as the one relevant for solving the task. The fact that we observed a trend in this direction, and not toward a stable non-turner response classification, might be due to our more abstract answering modes of which none involved physical pointing, the most embodied form of answering. We considered our answering modes more in between the continuum spanned by physical pointing and verbal description with spatial language.

In a review, Gramann (2013) summarizes the evidence for separate spatial neural networks that underlie turner and non-turner response patterns and underlying egocentric and allocentric reference frames. Evidence shows that an egocentric reference frame is associated with increased activity in the caudate nucleus, posterior parietal cortex, and human motion complex (Andersen and Buneo, 2002; Wolbers et al., 2004; Etchamendy and Bohbot, 2007; Wolbers et al., 2007; Whitlock et al., 2008; Iaria et al., 2013), whereas an allocentric reference frame is associated with increased activity in the hippocampus, amygdala, parahippocampal, perirhinal, entorhinal, and orbitofrontal cortices (Bohbot et al., 2007; Iaria et al., 2013). Moreover, in a virtual path integration study using EEG, Lin et al. (2015) found an egocentric reference frame was associated with the parietal, motor, and occipital cortices with dominant perturbations in the alpha band and theta modulation in the frontal cortex. They also found an allocentric reference frame was associated with the retrosplenial complex (RSC) with performance-related desynchronization of the 8–13 Hz frequency band and synchronization in the 12–14 Hz band, supporting previous evidence that the RSC transforms egocentric and allocentric spatial information into the respective other spatial reference frame. There is some support for linking egocentric and allocentric reference frames, and their corresponding neural processes, to the turner and non-turner answering patterns, respectively. It remains an open question how non-mover and spinner prevalence might relate to underlying neural activities. Non-movers seem more closely related to non-turners in the sense that they both do not seem to incorporate heading changes into their responses and, similarly, spinners seem more closely related to turners because they do seem to incorporate heading changes. Future studies should examine if non-movers and spinners are also closely linked to non-turner and turner cognitive and neural processes, and if they might correlate with reduced spatial abilities.

### 4.4. Outlook

The search for gender differences may be a complicated quest since our results suggest an interaction with task and possibly ethnicity. Instead of directly searching for gender differences, future studies should focus on investigating these interactions and aim for demographically more diverse samples. Our work gives more evidence to the embodied reference frame conflict hypothesis of Avraamides et al. (2004) and Riecke (2008). However, further investigations are needed to determine if non-turner answers are reflecting the use of an object-to-object (allocentric) representation or the use of a self-to-object (egocentric) representation that is still aligned with the (unchanged) physical body orientation. A focused investigation of the turner and non-turner behavior over more trials without feedback, looking for a resolution of the hypothetical reference frame conflict might be fruitful. The influence of ethnicity on the strategy selection for triangle completion and point-to-origin tasks adds a new facet to the influence of individual proclivities, motivating more studies with demographically diverse samples to get a more complete picture.

## Author contributions

AK: substantial contributions to conception of work, acquisition, analysis and interpretation of data, critical revisions for important intellectual content, final approval of version to be published, and agreement to be accountable for all aspects of the work. DS: substantial contributions to conception of work, acquisition, analysis and interpretation of data, draft of the work, final approval of version to be published, and agreement to be accountable for all aspects of the work. BR: substantial contributions to conception of work, acquisition, analysis and interpretation of data, critical revisions for important intellectual content, final approval of version to be published, and agreement to be accountable for all aspects of the work.

### Conflict of interest statement

The authors declare that the research was conducted in the absence of any commercial or financial relationships that could be construed as a potential conflict of interest. The Review Editor CG declares that, despite sharing the same affiliation as the author DS, the review process was handled objectively.
